# Germ granules in development

**DOI:** 10.1242/dev.201037

**Published:** 2023-01-30

**Authors:** Laura Thomas, Andrea Putnam, Andrew Folkmann

**Affiliations:** HHMI and Department of Molecular Biology and Genetics, Johns Hopkins University School of Medicine, Baltimore, MD 21205, USA

**Keywords:** RNA, Condensate, Germ granule, Oocyte, Primordial germ cell, Sperm

## Abstract

A hallmark of all germ cells is the presence of germ granules: assemblies of proteins and RNA that lack a delineating membrane and are proposed to form via condensation. Germ granules across organisms share several conserved components, including factors required for germ cell fate determination and maintenance, and are thought to be linked to germ cell development. The molecular functions of germ granules, however, remain incompletely understood. In this Development at a Glance article, we survey germ granules across organisms and developmental stages, and highlight emerging themes regarding granule regulation, dynamics and proposed functions.

## Teaser

**Summary:** A survey of germ granules across organisms and developmental stages, highlighting emerging themes regarding granule regulation, dynamics and proposed functions.

**Figure:**
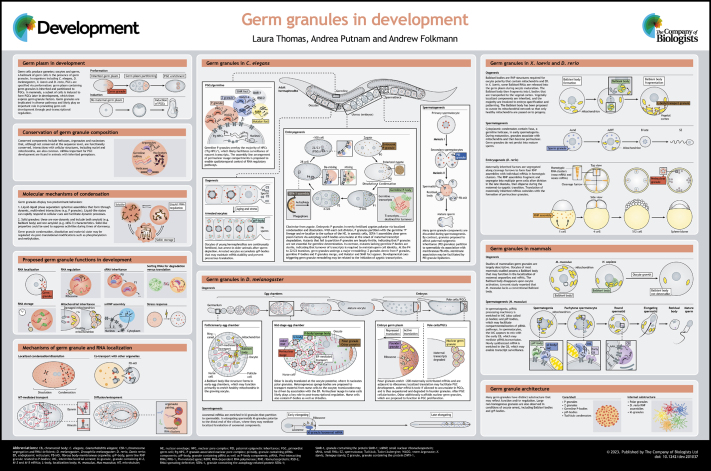


## Introduction

In sexually reproducing organisms, specialized cells called germ cells undergo meiosis to produce gametes, oocytes and sperm, which subsequently fuse to create a zygote. Germ cells contain unique structures known as germ granules that concentrate hundreds of RNAs and RNA-binding proteins, including conserved factors such as small RNA (sRNA) machinery, DEAD-box helicases and mRNAs that are crucial for primordial germ cell (PGC) development (Voronina et al., 2011). PGCs are specified in early development through two different mechanisms: preformation and induction (Hansen and Pelegri, 2021; Strome and Updike, 2015). In organisms that use the preformation mechanism, including *C. elegans*, *D. melanogaster*, *X. laevis* and *D. rerio*, germ granules assemble in a specialized cytoplasm called germ plasm that is transmitted from oocytes to embryos and asymmetrically partitioned to PCGs. In other organisms, including mammals, PGCs are induced later in development from undifferentiated progenitors, and germ granule components are expressed *de novo* in newly specified PGCs. Germ granules are therefore a prominent feature of organisms with either inherited germ plasm or induced PGCs. Given the high enrichment of RNA-binding proteins and conservation across diverse organisms, germ granules are proposed to play important roles in RNA regulation to facilitate PGC specification, formation and protection. In this article, we use ‘germ granule’ as a generic, catch-all term for RNA granules (Table 1) unique to germ cells, with the understanding that these comprise different granule types characterized by distinct compositions, including perinuclear granules characteristic of PGCs and immature germ cells, and cytoplasmic granules found in gametes and embryos.

**Table 1.**
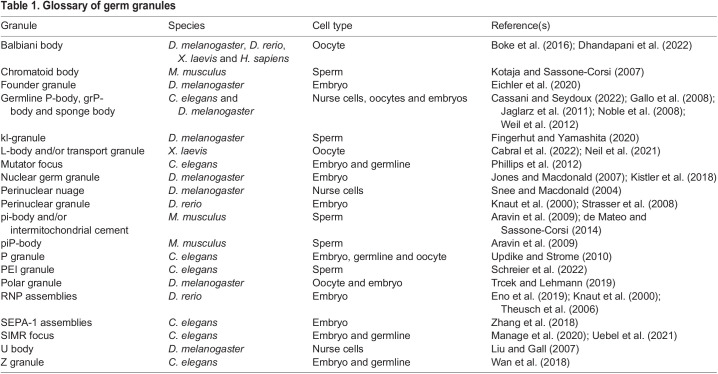
Glossary of germ granules

### Potential functions for germ granules in development

Diverse germ granule functions have been proposed based on analysis of granule composition, localization and dynamics, as well as genetic experiments in model organisms. Key proposed functions include: localization of germ cell determinants (Voronina et al., 2011), post-transcriptional RNA regulation (Eichler et al., 2020; Sheth et al., 2010; Updike et al., 2014), epigenetic inheritance and sRNA amplification (Ishidate et al., 2018; Schreier et al., 2022; Wan et al., 2018), sequestration/compartmentalization of RNA degradation, sRNA processing and translation activities (Eichler et al., 2020; Aravin et al., 2009; Wan et al., 2018), storage and protection of translationally repressed mRNAs (Hubstenberger et al., 2013; Jud et al., 2008; Noble et al., 2008), mitochondrial inheritance (Bilinski et al., 2017; Trcek and Lehmann, 2019), assembly and storage of uridine-rich small nuclear ribonucleoproteins (U snRNPs) (Liu and Gall, 2007), and responding to environmental stresses (Buckingham and Liu, 2011; Jud et al., 2008; Lee et al., 2020; Snee and Macdonald, 2009).

However, in most cases, germ granule function is still speculative. A key challenge in assigning function is that germ granule proteins, although concentrated in granules, often also exist at lower concentrations in the cytoplasm, making it challenging to uncouple germ granule-specific functions from the activity of soluble proteins. Methods to visualize biochemical activities *in vivo* will be needed to demonstrate granule-specific function. In the case of germ granules that form in specific locations in oocytes and embryos, *in situ* hybridization to visualize granule RNAs has provided strong support for a role in mRNA localization and germ cell fate specification. For example, in *D. melanogaster*, mislocalization of the granule nucleator Oskar leads to ectopic enrichment of mRNAs encoding germ cell fate regulators and, thus, ectopic induction of PGCs (Ephrussi and Lehmann, 1992).

### Material properties of germ granules

Germ granules belong to a class of cellular structures known as biomolecular condensates. Condensates are membraneless assemblies that lack a defined stoichiometry and concentrate biomolecules, most commonly proteins and nucleic acids (Banani et al., 2017). Material properties of biomolecular condensates are distinct from the surrounding cytosol, including higher viscosity, which can either enrich or exclude molecules in a size and property-dependent manner (Nott et al., 2016; Updike et al., 2011). Here, we focus on condensates that are specific to the germline; however, germ cells also contain condensates found in somatic cells, including stress granules, P-bodies and nucleoli (reviewed by Banani et al., 2017). The molecular mechanisms of condensate assembly are still under active investigation and studies of germ granules have played crucial roles in advancing this field. Two predominant mechanisms to describe the behavior of condensates were first described for germ granules.

Liquid-liquid phase separation (LLPS), first described for P granules in *C. elegans*, is proposed to play a role in the formation of many condensates (Brangwynne et al., 2009). LLPS is a process by which a solution of polymeric molecules spontaneously de-mixes into dense and dilute phases when above a critical concentration. The dense phase has liquid-like properties and enriches specific biomolecules, allowing the dense phase to function as a compartment (Alberti et al., 2019). LLPS is driven by dynamic, multivalent interactions between biomolecules, involving specific binding motifs in proteins and RNA (Banani et al., 2017; Li et al., 2012). Unlike membrane-bound structures, biomolecules in the dense phase can dynamically exchange with the dilute phase and are sensitive to environmental and biological changes, including temperature and post-translational modifications (PTMs) (Brangwynne, 2013).

Some germ granules are built around non-liquid scaffolds that resemble amyloid protein aggregates. For example, Balbiani bodies in *X. laevis* oocytes are scaffolded by Xvelo, a mostly disordered protein that forms a non-dynamic amyloid-like mesh in a reconstituted system (Boke et al., 2016). Amyloids have historically been studied in the context of neurodegenerative disease and Balbiani bodies were the first condensate to be described as ‘physiological amyloids’ that assemble and disassemble as part of normal development (Boke and Mitchison, 2017). Solid, but non-amyloid, condensates have also been described in the context of P granules in *C. elegans*, where MEG-3, an intrinsically disordered RNA binding protein, forms RNA-rich clusters on the surface of the liquid core of P granules (Putnam et al., 2019).

Key questions for the field involve understanding how the material properties of a condensate arise from the assembly of individual biomolecules and whether these material properties execute specific cellular tasks. Liquid-like material states can dynamically respond to cellular cues, enabling them to respond to environmental changes that occur on short time scales in early embryogenesis (Wang et al., 2014). In contrast, solid-like material properties could be used to suppress activities and protect biomaterials during times of dormancy, as oocytes can exist for months to years before fertilization (Jamieson-Lucy and Mullins, 2019). *In vitro* studies have highlighted that liquid-like condensates can mature over time to become more solid (Alberti and Hyman, 2016; Jawerth et al., 2020), suggesting that cells may have active processes to prevent maturation. In support of this idea, loss of the helicase CGH-1 results in the transition of the grP-body component CAR-1 into a solid lattice in the *C. elegans* germline (Hubstenberger et al., 2013). Future studies are needed to explore the contribution of material state to biological function.

### The role of RNA in germ granule dynamics

Nearly all identified germ granules contain RNA and RNA likely plays a crucial role in germ granule formation and material properties. *In vitro* systems have shown that, independently of protein, RNA can condense into liquid, gel-like or solid structures depending on sequence and length (Jain and Vale, 2017; Tauber et al., 2020; Van Treeck et al., 2018). Several perinuclear condensates, including Mutator foci and P granules in *C. elegans*, and the chromatoid body of mammalian sperm, assemble near nuclear pore complexes and disassemble when transcription is blocked (Lehtiniemi and Kotaja, 2018; Sheth et al., 2010; Uebel et al., 2020), consistent with a role for nascent transcripts in granule assembly. Condensation of germ granule proteins *in vitro* is often sensitive to RNA concentration. For example, RNA enhances the condensation of the P granule protein PGL-3 (*C. elegans*) and the L-body protein PTBP3 (*X. laevis*) (Cabral et al., 2022; Saha et al., 2016), and at high concentrations can also prevent condensation of the P granule protein MEG-3 (*C. elegans*) (Lee et al., 2020).

Factors that support mRNA production prevent the solidification of grP-bodies in *C. elegans* oocytes, suggesting a role for RNA in maintaining granule proteins in a liquid state (Hubstenberger et al., 2013). Similarly, in a reconstituted system, short RNAs decrease the viscosity and increase internal dynamics of condensates formed by the P granule helicase LAF-1 (Elbaum-Garfinkle et al., 2015).

### Post-translational modification of germ granule proteins

PTMs have emerged as a versatile mechanism for the spatiotemporal regulation of both somatic condensates and germ granules (Hofweber and Dormann, 2019; Schisa and Elaswad, 2021). Condensation is exquisitely sensitive to the valency of interactions and PTMs can modify valency by creating or occluding binding sites. Moreover, the combinatorial effects of multiple PTMs may act as a tunable mechanism to regulate condensate dynamics. Phosphorylation is a reversible modification where kinases and opposing phosphatases cooperate to regulate condensates. The kinase DYRK3/MBK-2 and phosphatase PP2A play crucial roles in the asymmetric polarization of P granules during embryonic development (Wang et al., 2014). Phosphorylation of MEG proteins by MBK-2 promotes P granule disassembly; additionally, MBK-2-mediated phosphorylation fluidizes the core protein PGL-3 to enable both efficient growth and regulated dissolution of P granules (Folkmann et al., 2021; Wang et al., 2014).

Many germ granule components contain arginine (R)-glycine (G) repeats (e.g. RGG- or RG-rich motifs) that are targeted for methylation (Anne et al., 2007; Kirino et al., 2010; Roovers et al., 2018). Unlike phosphorylation, arginine methylation is a low-dynamic modification and is thought to promote assembly, rather than dissolution, of germ granules. Methylated arginines are recognized by Tudor-domain proteins (Pek et al., 2012). In *D. melanogaster*, the methyltransferase Capsuléen promotes condensation of Vasa, Tudor and Maelstrom in the nurse cell nuage and facilitates assembly of the oocyte pole plasm via methylation of Sm proteins (Anne et al., 2007). Similarly, in *D. rerio*, methylation is suggested to promote condensation of Bucky ball to form the Balbiani body by generating binding sites for the Tudor domain-containing protein Tdrd6 (Roovers et al., 2018).

### Germ granule architecture

High-resolution imaging has revealed that many germ granules are multilayered and in fact correspond to a collection of condensates with distinct composition and material properties (Fare et al., 2021). A core/shell structure has been observed for embryonic P granules, Z granules, SEPA-1 assemblies, piP bodies and *D. melanogaster* Tudor/Aubergine condensates (Aravin et al., 2009; Vo et al., 2019; Wan et al., 2021; Wang et al., 2014; Zhang et al., 2018). For both P granules and Z granules, disruption of the shell-forming protein increases condensate size and decreases condensate number (Folkmann et al., 2021; Wan et al., 2021), and also affects the material properties of the condensates (Folkmann et al., 2021; Wan et al., 2021; Zhang et al., 2018). In the case of P granules, the shell-forming protein MEG-3 forms solid clusters that modulate condensate size by decreasing surface tension and recruits the kinase DYRK3/MBK-2 to fluidize the P granule core (Folkmann et al., 2021). In the examples described above, the substructure likely plays a key role in condensate function and/or regulation. In other cases, however, substructure may be a consequence of assembly, as proposed for the core/shell architecture of stress granules (Jain et al., 2016).

RNAs have non-homogeneous distributions in germline condensates, including polar granules in *D. melanogaster* and RNP assemblies in *D. rerio* (Eno et al., 2019; Trcek et al., 2015). RNAs in polar granules are organized in homotypic clusters with distinct spatial positioning relative to Vasa-protein condensates (Trcek et al., 2020). Whereas localization to polar granules requires specific RNA regions, the mechanism driving formation of homotypic clusters appears to involve the entire mRNA in a sequence-independent manner.

Non-homogenous distribution has also been noted for proteins, such as Vasa in polar granule precursors and Xvelo in Balbiani bodies (Boke et al., 2016; Jaglarz et al., 2011; Vo et al., 2019). High-resolution imaging studies of condensate components are likely to reveal additional condensate substructures; however, for most germ granules, the assembly, regulation and function of substructure remain unknown.

### Germ granule interactions with other granules and membranous organelles

A recurring feature for many germ granules is their ability to dock with other condensates. A prominent example is highlighted in the perinuclear nuage of *C. elegans*, where sRNA biogenesis factors form multi-condensate assemblages that include P granules, Mutator foci, Z granules and SIMR foci (Manage et al., 2020; Phillips et al., 2012; Wan et al., 2018). Strikingly, each nuage condensate contains a distinct set of proteins involved in sRNA regulation. Docking of germ granule condensates also occurs in spermatogenesis, where piP-bodies associate with pi-bodies to act in related steps of sRNA processing (Aravin et al., 2009). It is intriguing to speculate that such condensate interactions may partition sRNA processing steps to enable organization of pathway intermediates. Future studies are needed to determine the biological significance of condensate docking and the molecular rules that dictate the formation of these condensate assemblages.

Germ granules also contact membrane-bound organelles, including mitochondria, the nucleus, the endoplasmic reticulum and the Golgi. Although some interactions may be due to the crowded nature of the cytoplasm or to the general affinity of condensates for membranes, several of these interactions have clear functional relevance. For example, germ granules across diverse organisms associate stably with the nucleus, often in regions with highly clustered nuclear pore complexes (Voronina et al., 2011). Association with nuclear pore complexes likely facilitates germ granule surveillance of transcripts as they emerge from the nucleus, as nascent transcripts accumulate in perinuclear P granules and the chromatoid body (Sheth et al., 2010; Söderströmm and Parvinen, 1976).

In some cases, interactions with membranous organelles may mediate germ granule localization. For example, interactions with the endoplasmic reticulum have been speculated to mediate translocation of sponge bodies from nurse cells to the oocyte (Jaglarz et al., 2011), and PEI granules may ‘hitchhike’ on fibrous body-membranous organelles (FB-MOs) to be partitioned during spermatogenesis (Schreier et al., 2022). Germ granules across species and developmental stages commonly associate with mitochondria. As mitochondria are maternally inherited, enrichment of healthy mitochondria in the Balbiani body and *D. melanogaster* germ plasm may prevent passage of damaged mitochondria (Bilinski et al., 2017). Additionally, interaction with mitochondria has been proposed to mediate nucleation of intermitochondrial cement in sperm (Huang et al., 2011; Watanabe et al., 2011). Association with membranes may lower the threshold for condensation, as membrane surfaces restrict protein diffusion to a two-dimensional surface (Snead and Gladfelter, 2019).

## Conclusions and perspectives

Germ granules were originally observed by electron microscopy or cytochemistry as amorphous granulo-fibrillar structures (Eddy, 1976; Guraya, 1979). Recent advances in microscopy have revealed an increasing number of diverse granules and it is likely that many germ granules remain to be discovered. Although condensation of biomolecules is a potentially exciting mechanism for the unique requirements of germ cells, the role of most germ granules is still speculative. Ongoing research to dissect the function of condensates from soluble proteins will be crucial. To address this challenge, it will be necessary to better understand the mechanisms of assembly and regulation of germ granules through PTMs and enzymes, including kinases and RNA helicases.

Studies of germ granules in model systems have revealed a remarkable diversity of granule architecture, dynamics, material properties and interactions with other cellular structures. These findings raise many exciting questions regarding the functional relevance of these features. For example, it is speculated that granule material state corresponds to function, yet this proposal has not been conclusively demonstrated. Similarly, although solid granules such as the Balbiani body represent physiological amyloids, whether misregulated condensation leads to disease remains incompletely understood. For many germ granules, the function of elaborate substructure and granule-granule interactions remains unclear; indeed, there is no clear consensus as to what is considered a distinct granule versus the substructure of the same granule.

Finally, although model systems have significantly advanced our understanding of germ granule regulation, studies in mammals remain largely descriptive. Given the conservation of many granule components across species, an important future goal will be to determine whether similar mechanisms regulate germ granules in mammals and organisms with induced PGCs.

## Poster

Poster
