# Supplementary figures and images for: Germ granules in development
Source: Development. 2023 Jan 30;150(2):dev201037. doi: 10.1242/dev.201037 (PMC10165536; doi:10.1242/dev.201037)

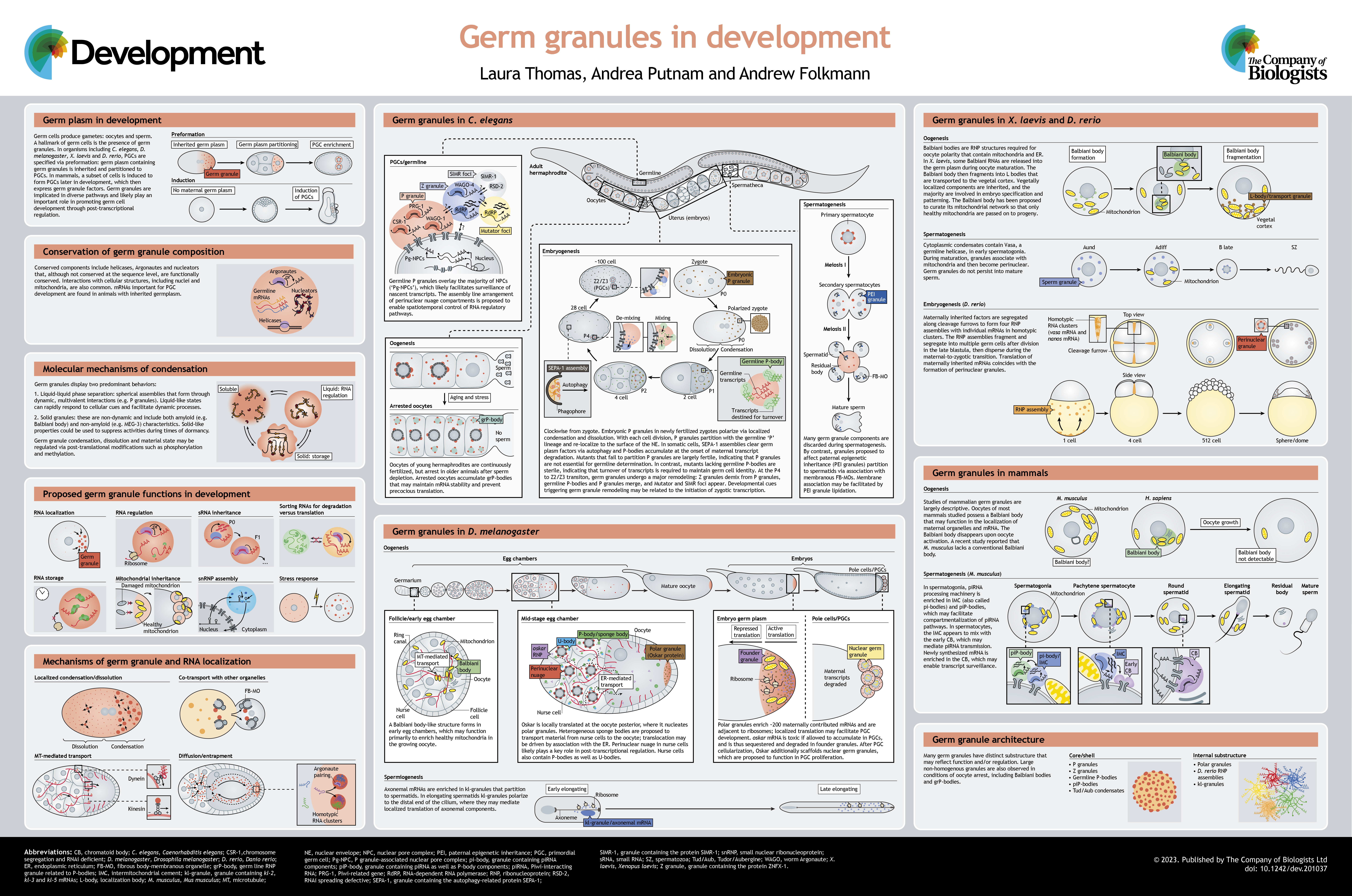

Supplement: Poster [file develop-150-201037-s1.jpg]
